# On the Nature of Chymus and Chylus

**Published:** 1801-09

**Authors:** 


### *On the Nature of Chymus and Chylus.*

THE nature of the *Chymus* and *Chylus*, and the changes which the firſt undergoes in the *inteſtina tenuia*, have not yet been ſufficiently examined, however intereſting a more accurate chemical analyſis of both ſubſtances may be for Phyſiologiſts. We are, therefore, much indebted to ſome able chemiſts and phyſicians, who have endeavoured to throw new light upon this ſubject by ſeveral chemical operations, the reſult of which, we ſhall communicate in the following lines. It is known from the experiments of HUNTER and SPALANZANI, that the ſtomach is endued with the faculty of making the milk and albuminous matter coagulate in a very ſhort ſpace of time, a phenomenon which is generally derived from an acid in the ſtomach, though ſeemingly contradictory to the later experiments of PARMENTIER and DEYEUX, (*Experiences et Obs. sur les differentes Especes de Lait. Straſb.* 7. *p.* 360), according to which the interior membrane of the ſtomach retained the property of coagulating the milk, even after having previouſly been treated with alkalis. The chymus of animals, however, ſhows moſt evident traces of an acid, particularly in the carnivorous tribe, which ſeems not to be very volatile, becauſe, being expoſed to the air, and boiled with water, it by no means loſes the property of reddening blue vegetable tinctures. It neither coagulates in the air nor by the fire, and after being inſpiſſated, it may be again diſſolved in water. Mr. SPALANZANI imagines, that the acid of the chymus originates from the remainders of the food, which opinion, however, is inconſiſtent with later experiments, according to which, the ſuccus gaſtricus and inferior membrane of the ſtomach have always been found found to be of an acid nature in animals that were purpofely fuffered to hunger, and being killed fome time after, the moft inconteftable traces of an acid were difcovered in their ftomachs.

The action of the fuccus gaftricus feems to confift, according to Profeffor AUTENRITH of Tubingen, in an oxydation, through means of which the albuminous fubftances of the ingefta coagulate, and are again diffolved by the acid and falts of the fuccus gaftricus. The juice that is found in the duodenum and inteftina tenuia contains particles coagulating in the air, and poffeffes at the fame time very little or no acidity, which is likewife not to be difcovered in the contents of the inteftina craffa. Chymus, and the contents of the inteftina tenuia in herbivorous and carnivorous animals differ, therefore, by the latter being not acid, and by their coagulating in fire and in the air. The contents of the inteftina craffa agree with chymus by not coagulating, though they differ from it by fhewing not the leaft traces of an acid. It has, however, been remarked by VAUQUELIN, that the excrements of men contain an acid fimilar to the acetous acid. (FOURCROY *Syfteme des Connaissances Chimiques*, *T. X. p.* 70. *A.* 312.)

Chyle has the appearance of a thin milk, becomes whiter in the air, and coagulates only in part; it is likwife deprived of all oily particles. It differs from lymph by being milky.

We fhall now enter upon the queftion, how the chymus is converted into chyle, which proceeding takes place in the duodenum, and is thought to be effected by the bile; in order to afcertain which, the chymus of carnivorous and herbivorous animals was mixed with bile, from which a precipitation was produced, and a white cuticule arofe on the furface, a phenomenon that even takes place when the chyle and bile of different animals are mixed together. No precipitation is occafioned by the gall in the contents of the inteftina craffa. Although this white precipitate feems to have been pre-exiftent in the chyle, yet one may fuppofe it to originate from the albuminous part of the gall, which is feparated by the acid of the chymus, becaufe a fimilar precipitation enfued, on adding vinegar to pure gall. In order to afcertain this circumftance, 27 grains of the chyle of a cat were mixed with 14 gr. of bile; the precipitate thus obtained, weighed about 3 $\frac{1}{4}$ gr. To 18 gr. of bile taken from the fame cat, 27 gr. of vinegar were added, by which a precipitate was produced, weighing only 2 $\frac{2}{3}$ gr. when dry. 4 fcruples of chymus of a cat precipitated in *one* drachm of gall about 9 gr. whereas 4 fcruples of alkohol only precipitated 3 $\frac{1}{7}$ gr. from the fame dofes of gall. It feems to refult from thefe experiments, that the above mentioned precipitation is is owing both to the albuminous parts of the gall, as well as to the chymus.

For explaining the manner in which the chymus is changed into chyle, it may be adopted, that it proceeds from an abforption of oxygen, and the procefs of digeftion is accordingly performed in the following fucceffion. The albuminous parts of the food coagulate in the ftomach by an oxygenation, and are again diffolved by the acid of the fuccus gaftricus, but partly changed into a milky emulfion. The whole mafs is now deprived of its oxygen by the addition of the gall, whereby the lymphatic parts of the gall and of the food coagulate in fmall particles, which are again changed into a mucilaginous mafs, by a greater defoxydation, which poffeffes the property of coagulating on contact with the atmofphere, and after being abforbed by the lymphatic veffels, obtains the name of chyle.

According to the experiments of Dr. Reuss and Emmert, the chyle has more analogy with blood than with milk, on which account it rather deferves the appellation of white blood than of milky juice. It neither contains oily particles nor faccharum lactis, the characteriftic conftituents of milk. We may alfo conjecture, that the phofphat of the oxyd of iron, which, according to the interefting difcovery of Fourcroy and Vauquelin, is the colouring matter of the blood in the ftate of fuperoxydation, does pre-exift in the chyle in form of a white phofphat. (Ch. L. Werner, *Experimenta circa modum quo Chymus in Chylum mutatur, in Animalibus instituta præside* J. H. F. Autenrith, Prof. Tubingenfi.)

